# Discoidin domain receptor 1 deficiency in vascular smooth muscle cells leads to mislocalisation of N-cadherin contacts

**DOI:** 10.1242/bio.041913

**Published:** 2019-07-30

**Authors:** Songyi Xu, Sudarshan Bala, Michelle P. Bendeck

**Affiliations:** 1 Laboratory Medicine and Pathobiology, Faculty of Medicine, University of Toronto, Toronto, Ontario M5G 1M1, Canada; 2 Translational Biology and Engineering Program, Ted Rogers Centre for Heart Research, Toronto, Ontario M5G 1M1, Canada

**Keywords:** DDR1, N-cadherin, Vascular smooth muscle cells, Lipid rafts, Cell–cell contacts

## Abstract

N-cadherin mediates cell–cell contacts in vascular smooth muscle cells (VSMCs), and regulates VSMC behaviours including migration and proliferation. Discoidin domain receptor 1 (DDR1) is a collagen binding receptor also implicated in these processes. Previous studies have shown that both N-cadherin and DDR1 are upregulated after vascular injury, but it is not known whether there is a relationship between the two molecules. In the current study we found that N-cadherin was mislocalised from cell–cell junctions in the absence of DDR1. This occurred in spite of the fact that there was no significant difference in total cell lysate levels of N-cadherin between DDR1+/+ and DDR1−/− VSMCs. Analysis of lipid raft fractions revealed decreased N-cadherin and associated junctional complex catenins in DDR1−/− compared to DDR1+/+ VSMCs. Treatment with cholesterol oxidase or methyl-β-cyclodextrin to disrupt lipid rafts removed N-cadherin and DDR1 from the raft fractions. Reciprocal co-immunoprecipitations suggested the association of DDR1 and N-cadherin. Importantly, transfection of DDR1−/− cells with full-length DDR1b rescued the formation of N-cadherin junctions. Together, these data reveal that N-cadherin cell–cell contacts in VSMCs are regulated through interactions with DDR1 and both molecules are located in lipid rafts.

## Abstract (precis)

**Summary:** Here we show for the first time that discoidin domain receptor 1 associates with and stabilizes N-cadherin cell–cell contacts in vascular smooth muscle cells.

## INTRODUCTION

Atherosclerosis is a leading cause of death worldwide (Bennett et al., 2016). The atherosclerotic lesion is characterized by the development of fibroinflammatory lipid plaques in the inner lining of arteries (Xu et al., 2015). This involves the directed migration of vascular smooth muscle cells (VSMCs) from the medial to the intimal layer of the vessel wall, where they undergo proliferation and synthesize extracellular matrix to form a fibrous cap that covers the atherosclerotic plaque (Libby et al., 2011). VSMCs are also the predominant cell type found in the lesions during restenosis after angioplasty, stenting and in vein graft disease, where they migrate and proliferate to form the thickened neointima (Davies and Hagen, 1994). It is well established that soluble growth factors regulate VSMC proliferation and migration (Louis and Zahradka, 2010). Moreover, within the vessel wall, VSMCs make adhesive contacts with neighbouring cells as well as the extracellular matrix. The dynamic remodelling of cell–cell and cell–matrix contacts can regulate cellular and molecular processes in VSMCs to coordinate cell behaviours such as proliferation and migration (Gerthoffer, 2007; Johnson, 2014; Louis and Zahradka, 2010).

Balloon denudation of the endothelium is a model of mechanical injury to the artery that triggers the directional migration of VSMCs to form a thickened neointimal layer (Davies and Hagen, 1994). Following mechanical injury of the carotid artery in the rat, our lab showed elevated expression of both discoidin domain receptor 1 (DDR1) (Hou et al., 2001) and N-cadherin in VSMCs (Jones et al., 2002). DDR1 is a collagen-binding receptor tyrosine kinase, and its deletion reduces VSMC migration towards collagen *in vitro* and attenuates neointimal thickening and atherosclerotic plaque formation *in vivo* (Franco et al., 2008; Hou et al., 2001). Recent research has shown that DDR1 can stabilize cadherin-containing contacts, but many studies have focused on the effects of DDR1 in stabilizing E-cadherin contacts in epithelial cells (Chen et al., 2016; Eswaramoorthy et al., 2010; Yeh et al., 2011). Furthermore, these effects were found to be context-dependent. In normal epithelial cells, DDR1 forms a complex with E-cadherin, stabilizing cell–cell adhesions (Eswaramoorthy et al., 2010; Yeh et al., 2011). By contrast, in cancer, DDR1 is upregulated and promotes epithelial-mesenchymal transition (EMT) by increasing the expression of N-cadherin, promoting cell migration and invasion (Azizi et al., 2019; Huang et al., 2016; Miao et al., 2013; Shintani et al., 2008). Clearly, the effects of DDR1 on cadherin-based contacts cannot be extrapolated between different cell types and conditions. To the best of our knowledge, there has been no research studying the effects of DDR1 on N-cadherin cell–cell contacts in VSMCs.

VSMCs express several types of cadherin molecules, including N-cadherin, T-cadherin, R-cadherin, FAT1-cadherin and OB-cadherin (Resnik et al., 2009; Xu et al., 2015). OB-cadherin promotes cell–cell adhesion and collectivization of VSMCs (Balint et al., 2015). T-cadherin (Ivanov et al., 2004) stimulates proliferation and induces migration of VSMCs, potentially contributing to intimal hyperplasia in atherosclerotic lesions and vessel stenosis. FAT1- (Hou et al., 2006) and R-cadherin (Slater et al., 2004) may have an antiproliferative function through the sequestration of β-catenin, preventing its translocation to the nucleus to activate cyclin D1. FAT1-cadherin increases cell–cell adhesive force and reduces migration and invasion in epithelial cells (Hu et al., 2018).

Previous research from our lab showed that N-cadherin was the most abundant cell–cell adhesion molecule expressed by VSMCs, and that it played an important role in regulating directional migration (Sabatini et al., 2008). Specifically, in mechanical wounding experiments performed *in vitro*, the disruption of N-cadherin contacts at the wound edge led to a polarized posterior-lateral distribution of N-cadherin at the cell periphery, and this was required for the directional migration of VSMCs into the wound (Sabatini et al., 2008). This migratory response was inhibited by treatment with either N-cadherin blocking antibody or peptide, which bind to N-cadherin molecules on the cell surface to inhibit its function (Sabatini et al., 2008). Consistent with our findings, George and colleagues reported that inhibiting N-cadherin using HAV peptides attenuated VSMC migration and neointimal thickening in a human saphenous vein organ culture model (Lyon et al., 2010).

N-cadherin belongs to the family of classical cadherins, which are transmembrane cell–cell adhesion molecules that undergo calcium-dependent homophilic binding via the extracellular domain (Angst et al., 2001). The cytoplasmic domain of N-cadherin can bind to catenin molecules which interact with the actin cytoskeleton to mediate strengthening and stabilization of the junctional complex (Gumbiner, 2005). In addition, N-cadherin contacts are stabilized by association with lipid rafts, which are cholesterol/sphingolipid-enriched microdomains in the cell membrane. Lipid rafts facilitate the functional grouping of proteins during a wide range of processes including cell migration, intracellular trafficking, and signal transduction (Causeret et al., 2005; Resnik et al., 2011; Schinner et al., 2017). Little is known about the lipid raft localization of the N-cadherin adhesion complex or the DDR1 in VSMCs. The role of DDR1 in stabilizing N-cadherin contacts has not yet been investigated. Therefore, in this study, using VSMCs from a DDR1-knockout mouse model, we describe a novel mechanism by which DDR1 regulates N-cadherin cell–cell contacts in VSMCs.

## RESULTS

### N-cadherin cell–cell contacts were disrupted in DDR1−/− VSMCs

**Fig. 1.**
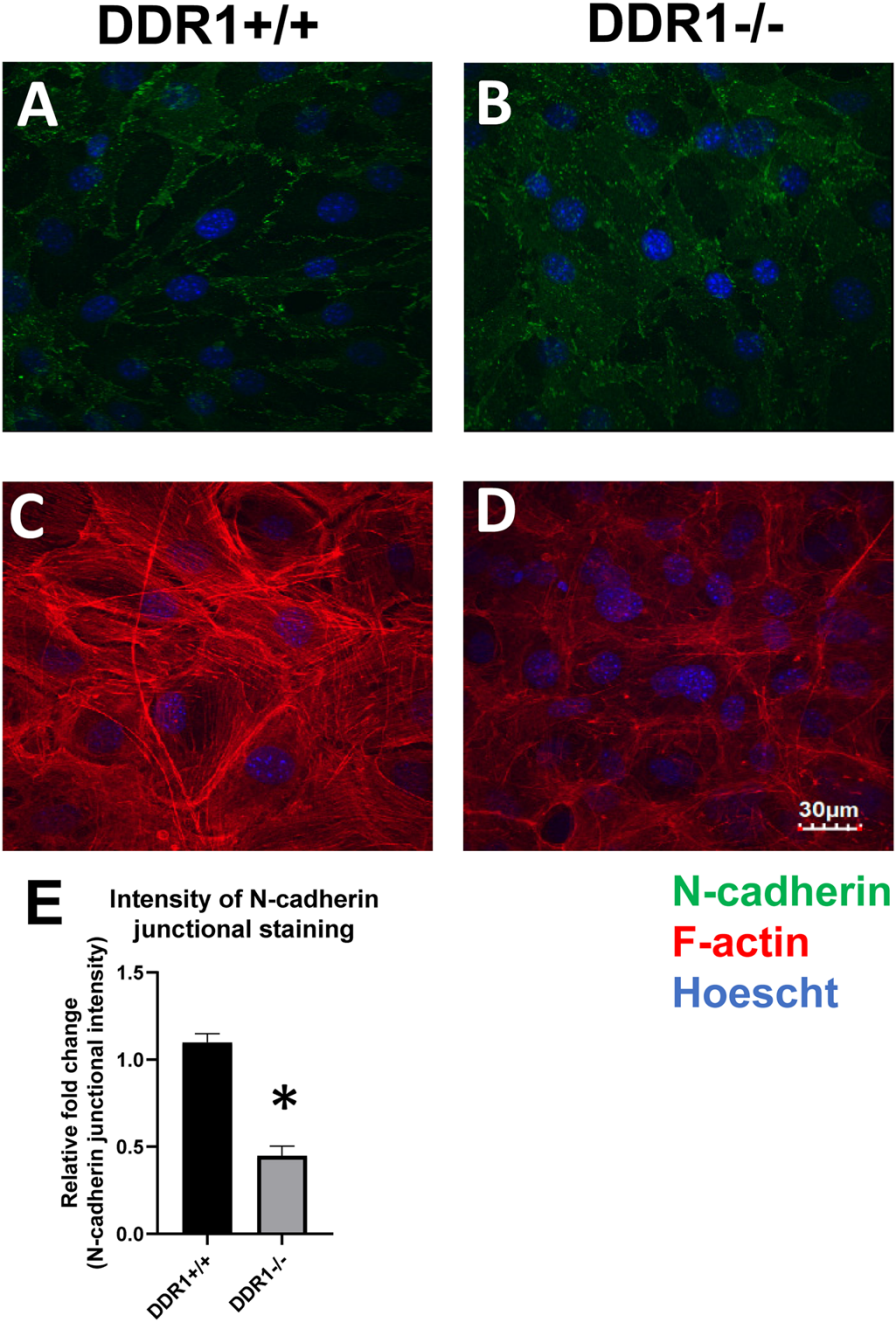
**N-cadherin cell–cell contacts were disrupted in DDR1−/− VSMCs.** (A–D) Immunostaining of N-cadherin (green; 610921 BD Biosciences) and F-actin (red; Alexa 568 phalloidin) in DDR1+/+ and DDR1−/− VSMCs. Nuclei are counterstained with Hoechst (blue). (E) Relative fold change in intensity of junctional N-cadherin staining in DDR1+/+ and DDR1−/− VSMCs. Experiment was repeated three times. Data are plotted as mean±s.e.m. Student’s *t*-test was performed. * indicates statistical significance of *P*<0.05.

**Fig. 2.**
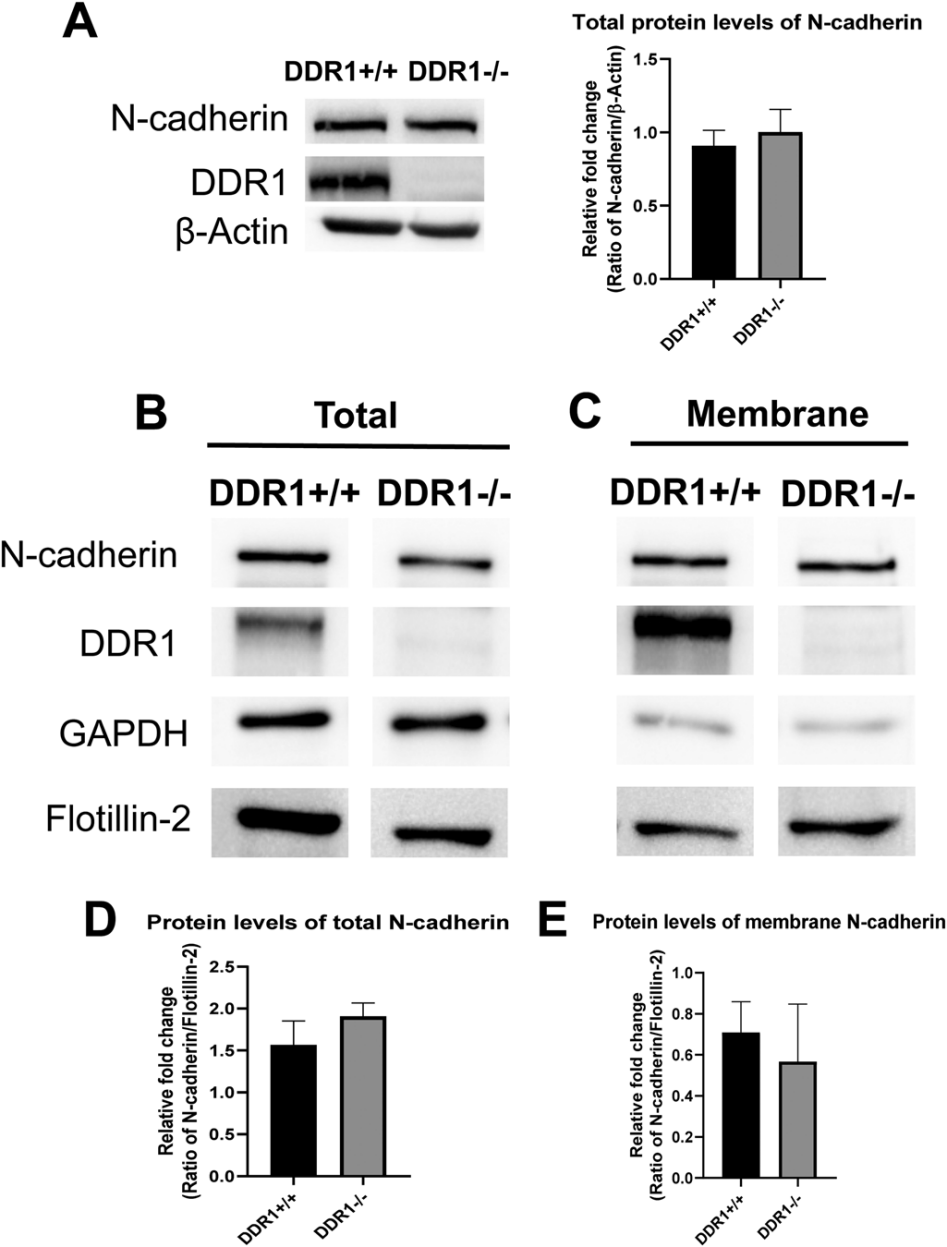
**There was no difference in total cell lysate or whole membrane N-cadherin protein levels between DDR1+/+ and DDR1−/− VSMCs.** (A) Total cell lysate of DDR1+/+ and DDR1−/− VSMCs, immunoblotted for N-cadherin, DDR1 and β-actin. Results were quantified as the level of N-cadherin normalized to β-actin, and expressed relative to DDR1+/+ VSMCs, and Student’s *t*-test was performed (*n*=3). Protein levels of N-cadherin and DDR1 in total cell lysate B, and whole membrane fractions C, of DDR1+/+ and DDR1−/− VSMCs. GAPDH and Flotillin-2 were used as controls. Blots were spliced to compose the images, but samples were immunoblotted and developed simultaneously for each blot. Blots in panels B and C were processed separately. Relative fold change in total cell lysate (D), and whole membrane (E), N-cadherin protein levels in DDR1+/+ and DDR1−/− VSMCs normalized to flotillin-2 and expressed relative to DDR1+/+ VSMCs (*n*=3). Data are plotted as mean±s.e.m. Student’s *t*-test was performed.

Primary DDR1+/+ and DDR1−/− VSMCs were grown to confluence, fixed and stained for N-cadherin to visualize cell–cell contacts. In DDR1+/+ VSMCs, N-cadherin staining was observed in a zipper-like conformation around the cell periphery (Fig. 1A). By contrast, in DDR1−/− VSMCs zipper-like structures were reduced and most N-cadherin staining was diffuse in the cytoplasm (Fig. 1B). Quantification of N-cadherin junctional staining demonstrated significantly higher (60% greater) N-cadherin staining intensity at cell–cell contacts of DDR1+/+ VSMCs compared to DDR1−/− VSMCs (Fig. 1E). While more intense F-actin staining and a greater number of stress fibres was observed in DDR1+/+ VSMCs (Fig. 1C), an intact actin network was still observed in DDR1−/− VSMCs (Fig. 1D), showing that overall cell integrity was not compromised by DDR1 deletion. Furthermore, there were no differences in cell viability or density between DDR1+/+ and DDR1−/− VSMCs. Immunoblotting whole cell lysates of DDR1+/+ and DDR1−/− VSMCs revealed similar levels of total N-cadherin protein in both genotypes (Fig. 2A). To determine whether the level of N-cadherin protein in the membrane was reduced in DDR1−/− SMCs, the membrane protein fraction was isolated by ultracentrifugation and probed with an antibody against N-cadherin (Fig. 2B Total, 2C Membrane). However, there were no significant differences in the total (Fig. 2D), or in the membrane protein levels (Fig. 2E) of N-cadherin between genotypes. Taken together, this data suggests that the major consequence of DDR1 deficiency is a reduction in the organization of N-cadherin into stable, morphologically distinct cell–cell junctions.

### N-cadherin co-immunoprecipitated with DDR1 and catenins

**Fig. 3.**
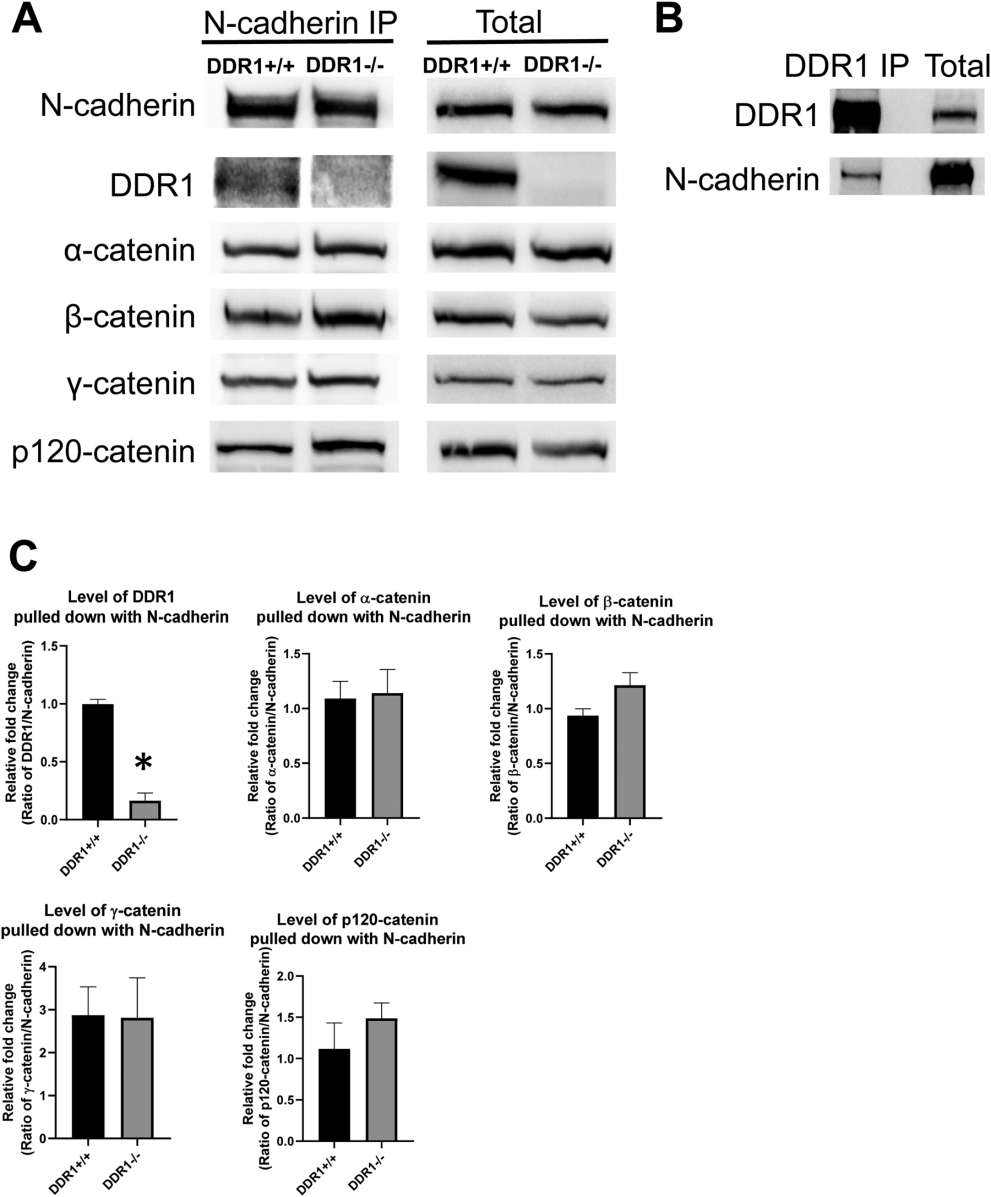
**N-cadherin co-immunoprecipitated with DDR1, α-, β-, γ-, and p120-catenin.** (A) Immunoprecipitation of N-cadherin or total cell lysate of DDR1+/+ and DDR1−/− VSMCs blotted for N-cadherin, DDR1, α-catenin, β-catenin, γ-catenin and p120-catenin. Blots were spliced to compose the image, but all samples were immunoblotted and developed simultaneously. (B) Immunoprecipitation of DDR1 or total cell lysate of DDR1+/+ VSMCs blotted for DDR1 and N-cadherin. Experiments were repeated three times. (C) Relative fold change of DDR1, α-catenin, β-catenin, γ-catenin, and p120-catenin levels (*n*=3) pulled down with N-cadherin in DDR1+/+ and DDR1−/− VSMCs was normalized to N-cadherin and expressed relative to DDR1+/+ VSMCs. Data are plotted as mean±s.e.m. Student’s *t*-test was performed. * indicates statistical significance of *P*<0.05.

Next we investigated whether DDR1 could associate with N-cadherin in VSMCs. DDR1 and N-cadherin were pulled down together using reciprocal co-immunoprecipitation (Fig. 3A,B). Catenins associate with the cytoplasmic tail of N-cadherin, facilitate interaction with the cytoskeleton, and strengthen the junctional complex (Angst et al., 2001). We performed co-immunoprecipitations to determine whether there were differences in N-cadherin association with various catenins comparing DDR1+/+ and DDR1−/− VSMCs. N-cadherin co-immunoprecipitated with α-, β-, γ-, and p120-catenins, and there were no differences in association comparing the two genotypes of VSMCs (Fig. 3A). Quantification of the western blots showed that the association of N-cadherin with catenins did not differ significantly between DDR1+/+ and DDR1−/− VSMCs (Fig. 3C).

### Cytoplasmic N-cadherin in DDR1−/− VSMCs was disrupted by Triton treatment

**Fig. 4.**
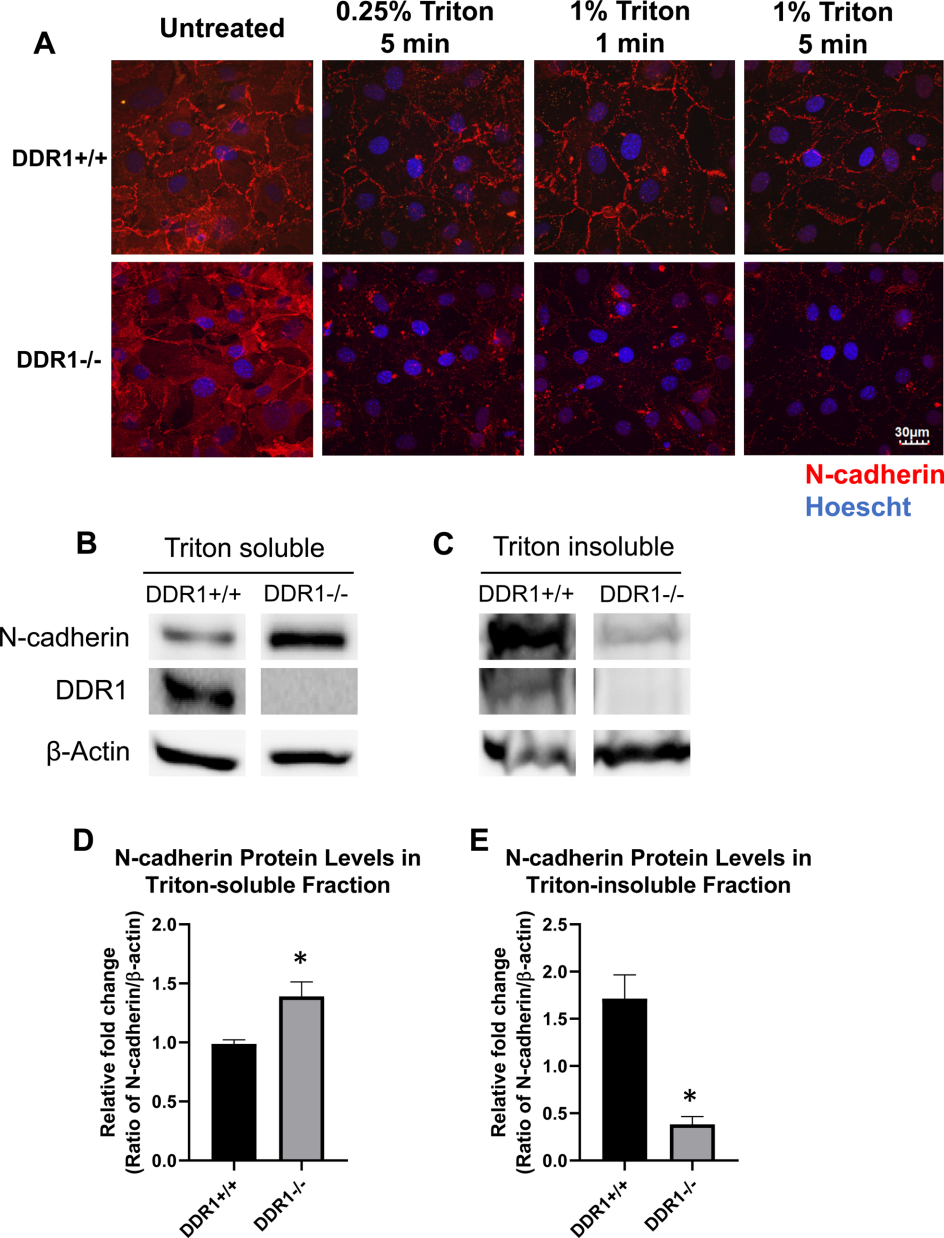
**N-cadherin in DDR1−/− VSMCs was disrupted by Triton treatment.** (A) Immunofluorescence of DDR1+/+ and DDR1−/− VSMCs treated with or without 0.25% or 1% Triton for 1 or 5 min on ice, fixed and stained for N-cadherin (red; 610921 BD Biosciences). Nuclei are counterstained with Hoechst (blue). Protein levels of N-cadherin, DDR1, and β-actin in triton-soluble (B), and triton-insoluble (C), fractions of cell lysate from DDR1+/+ and DDR1−/− VSMCs. Blots were spliced to compose the image, but all samples were immunoblotted and developed simultaneously. Blots in panels B and C were processed separately. Relative fold changes of triton-soluble (*n*=8) (D), and triton-insoluble (*n*=4) (E). N-cadherin protein in DDR1+/+ and DDR1−/− VSMCs normalized to β-actin, and expressed relative to DDR1+/+ VSMCs. Data are plotted as mean±s.e.m. Student’s *t*-test was performed. * indicates statistical significance of *P*<0.05.

To further probe the localization of N-cadherins in DDR1+/+ and DDR1−/− VSMCs, we performed Triton extraction to determine the cellular compartments in which the N-cadherin proteins reside. This takes advantage of the fact that proteins in the cytoplasm, during synthesis, and recycling are Triton soluble, while stable membrane proteins are Triton insoluble. In DDR1+/+ VSMCs, Triton treatment did not disrupt N-cadherin cell–cell contacts, which remained in a zipper-like configuration at the cell periphery (Fig. 4A). However, in DDR1−/− VSMCs, cytoplasmic N-cadherin staining was markedly reduced after Triton treatment, with more disruption occurring with increased concentration or time of Triton exposure (Fig. 4A). This suggests more N-cadherin was present in the Triton soluble pool in DDR1−/− VSMCs. To measure the amount of N-cadherin present in Triton soluble versus insoluble fractions, DDR1+/+ and DDR1−/− VSMCs were lysed in 1% Triton, and N-cadherin protein levels were assessed by western blotting (Fig. 4 B,C). DDR1−/− VSMCs showed significantly higher levels of N-cadherin in the Triton soluble fraction compared to DDR1+/+ cells (Fig. 4D). By contrast, the DDR1−/− cells had significantly less N-cadherin in the Triton insoluble fraction compared to DDR1 +/+ cells (Fig. 4E).

### Levels of N-cadherin and catenins were reduced in lipid rafts from DDR1−/− VSMCs

**Fig. 5.**
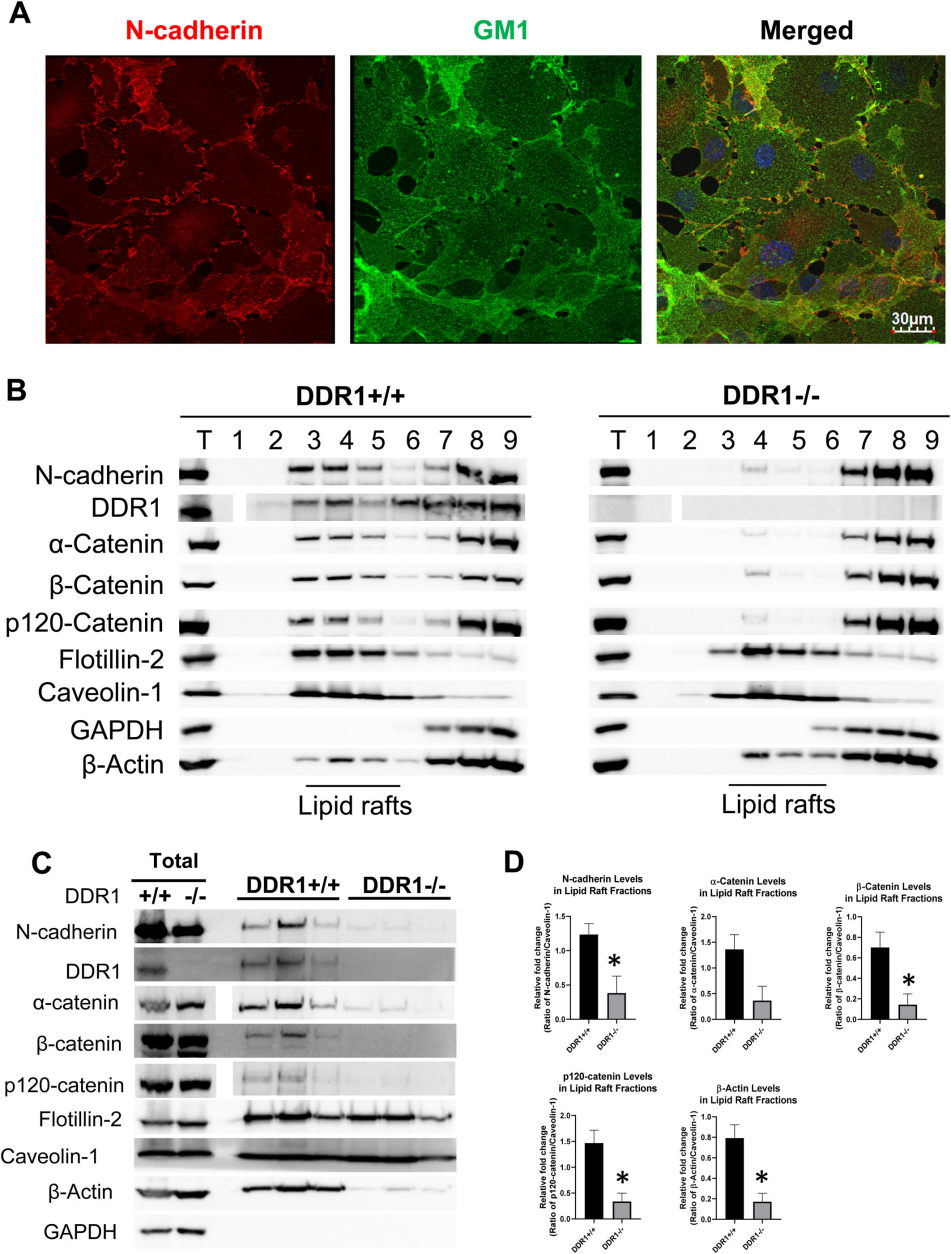
**Levels of N-cadherin and catenins were reduced in lipid rafts from DDR1−/− VSMCs.** (A) Immunofluorescence of DDR1+/+ VSMCs stained for N-cadherin (red; 610921 BD Biosciences) and GM1 (green; Alexa 488 cholera toxin B, C34775 Life Technologies). Nuclei are counterstained with Hoechst (blue). (B) Protein levels of N-cadherin, DDR1, α-catenin, β-catenin, p120-catenin, flotillin-2, caveolin-1, GAPDH, and β-actin in total cell lysate (T), and fractions (1–9) after separation of cell lysate from DDR1+/+ and DDR1−/− VSMCs along a 40%/30%/5% sucrose gradient. (C) Protein levels of N-cadherin, DDR1, α-catenin, β-catenin, p120-catenin, flotillin-2, caveolin-1, GAPDH and β-actin in total cell lysate and fractions 3–5. Blots were spliced to compose the image, but all samples were immunoblotted and developed simultaneously. (D) Relative fold changes of N-cadherin, α-catenin, β-catenin, p120-catenin and β-actin (*n*=3) in lipid raft fractions of DDR1+/+ and DDR1−/− VSMCs normalized to caveolin-1, expressed relative to DDR1+/+ VSMCs. Data are plotted as mean±s.e.m. Student’s *t*-test was performed. * indicates statistical significance of *P*<0.05.

Lipid rafts are sphingolipid- and cholesterol-rich microdomains in the cell membrane that can cluster and organize proteins into signalling complexes, and comprise a cell membrane structure that is resistant to Triton solubilisation (Lingwood and Simons, 2010). To determine whether N-cadherin cell–cell junctions associate with lipid rafts in wild-type VSMCs, we first labelled lipid rafts with fluorescent cholera toxin B that binds to ganglioside GM1, and then fixed and stained the cells for N-cadherin. N-cadherin and GM1 were co-localized at regions of cell–cell contact, and quantification revealed that approximately 2% of N-cadherin signal co-localized with GM1 (Fig. 5A). To compare the levels of N-cadherin protein in lipid rafts between DDR1+/+ and DDR1−/− VSMCs, cells were lysed in 1% Triton, lipid raft proteins were separated by ultracentrifugation on a sucrose density gradient, then fractions were subjected to western blotting. Staining with antibodies for the lipid raft markers flotillin-2 and caveolin-1 showed that lipid raft proteins were concentrated in fractions 3–5 (Fig. 5B). GAPDH, a cytoplasmic protein loading control, was absent from these fractions and present in fractions 6–9 (Fig. 5B). N-cadherin, DDR1, α-, β-, and p120-catenin were present in the lipid raft fractions from DDR1+/+ VSMCs, but markedly reduced in the lipid raft fractions of DDR1−/− VSMCs (Fig. 5B). Because proteins that associate with membrane lipid rafts constitute only a small fraction of the total proteins inside the cell, the majority of N-cadherin, DDR1 and catenin proteins were found in non-lipid raft fractions (fractions 7–9).

To facilitate better comparison of protein levels in the lipid raft fractions of DDR1+/+ and DDR1−/− VSMCs, the lipid raft fractions from several dishes were pooled and run side by side on an SDS-PAGE gel and analysed by western blotting (Fig. 5C). There were significant reductions in the levels of N-cadherin, α-, β, and p120-catenins in the lipid rafts from DDR1−/− VSMCs compared to DDR1+/+ VSMCs (Fig. 5D). β-actin levels were also reduced in the lipid raft fractions of DDR1−/− compared to DDR1+/+ VSMCs (Fig. 5C), likely due to reduced anchoring of N-cadherin to the membrane and cytoskeleton in the DDR1−/− VSMCs. There were no differences in levels of these proteins in total cell lysates between DDR1+/+ and DDR1−/− VSMCs (Fig. 5C).

### Disruption of lipid rafts resulted in the reduction of N-cadherin, catenins and DDR1

**Fig. 6.**
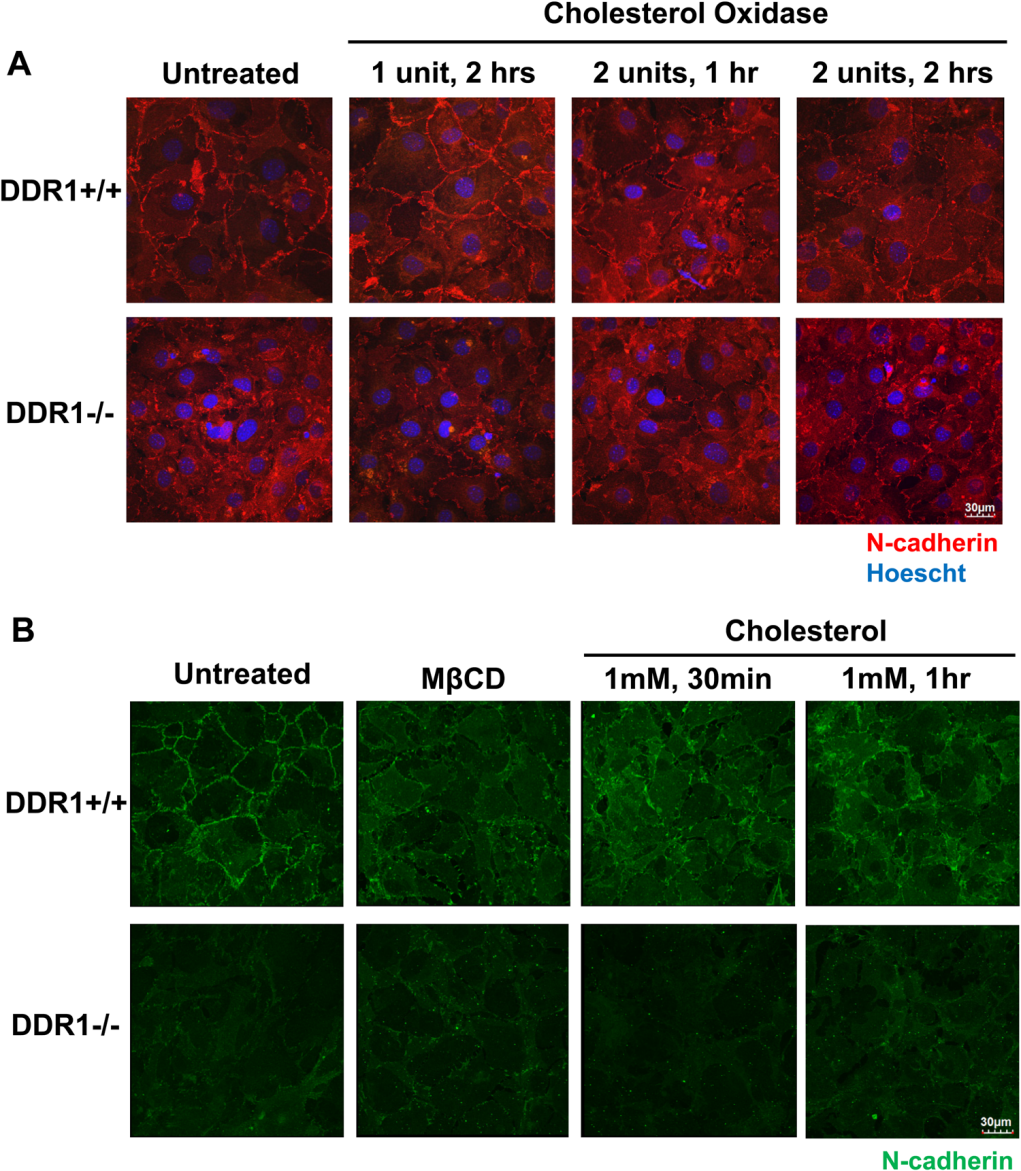
**Disruption of lipid rafts resulted in the reduction of N-cadherin contacts between cells.** (A) Immunofluorescence of DDR1+/+ and DDR1−/− VSMCs treated with or without 1 or 2 units of cholesterol oxidase for 1 or 2 h, fixed and stained for N-cadherin (red; 610921 BD Biosciences). Nuclei are counterstained with Hoechst (blue). (B) Immunofluorescence of DDR1+/+ and DDR1−/− VSMCs treated with or without 1 mM of MβCD for 30 min, followed by 1 mM of MβCD-cholesterol for 30 min or 1 h, fixed and stained for N-cadherin (green; 610921 BD Biosciences).

**Fig. 7.**
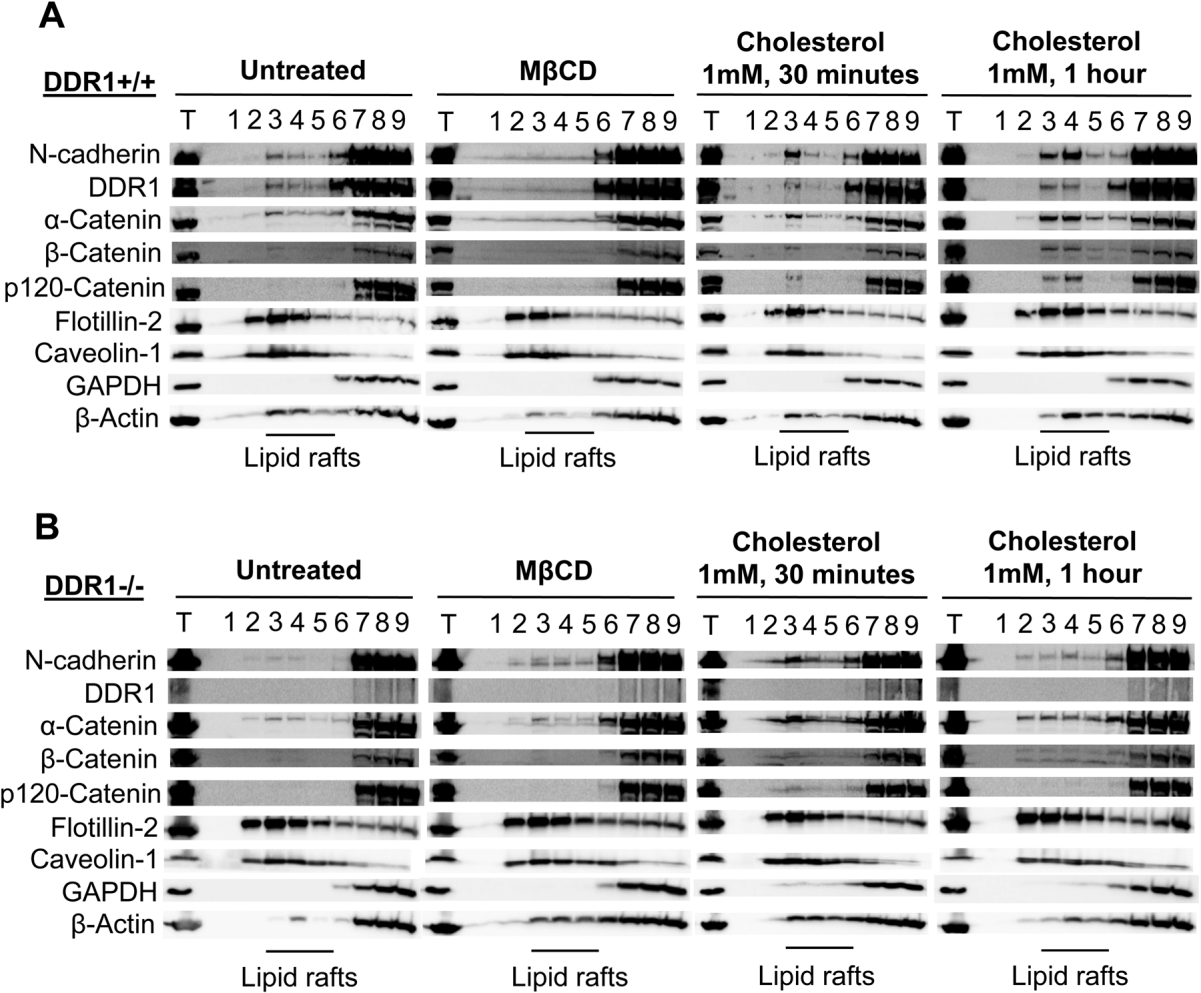
**Disruption of rafts resulted in reduction of N-cadherin, catenins and DDR1 in the lipid raft fraction.** Protein levels of N-cadherin, DDR1, α-catenin, β-catenin, p120-catenin, flotillin-2, caveolin-1, GAPDH and β-actin in total cell lysate (T) and fractions (1–9) after separation of cell lysate along a 40%/30%/5% sucrose gradient from DDR1+/+ (A), and DDR1−/− (B), VSMCs that were untreated or treated with 1 mM MβCD, followed by 1 mM of MβCD-cholesterol for 30 min or 1 h. Experiments were repeated three times.

Cholesterol oxidase and methyl-β-cyclodextrin (MβCD) disrupt lipid rafts by altering their cholesterol content, either through catalysing cholesterol catabolism or directly removing cholesterol molecules. Cells were treated with cholesterol oxidase or MβCD to disrupt lipid rafts, then stained for N-cadherin to observe the effects on cell–cell contacts. In untreated cells, N-cadherin contacts were arranged in a zipper conformation around the periphery of DDR1+/+ VSMCs, while N-cadherin staining was more diffuse in the cytoplasm of DDR1−/− VSMCs (Fig. 6A). Treatment with cholesterol oxidase disrupted peripheral, junctional N-cadherin staining in DDR1+/+ VSMCs, with dose and time dependent increases in disruption (Fig. 6A). By contrast, cholesterol oxidase treatment did not affect the diffuse cytoplasmic N-cadherin staining in DDR1−/− VSMCs (Fig. 6A). MβCD treatment had similar results, disrupting N-cadherin at cell–cell contacts of DDR1+/+ VSMCs with little effect on cytoplasmic N-cadherin staining in DDR1−/− VSMCs (Fig. 6B). The disruptive effect of MβCD on the cell membrane was rescued by treating the cells with exogenous cholesterol, which restored N-cadherin at cell–cell contacts in DDR1+/+ VSMCs (Fig. 6B). Quantification of N-cadherin junctional staining in these experiments revealed a decrease from 0.94±0.03 in untreated cells to 0.44±0.04 in MβCD treated cells. Subsequent rescue of the MβCD treated cells with exogenous cholesterol resulted in the restoration of junctional N-cadherin staining to control levels 0.96±0.08. These results were confirmed by analysing proteins in lipid rafts from MβCD treated cells. MβCD treatment led to the displacement of N-cadherin, the catenins and DDR1 from lipid raft fractions of DDR1+/+ VSMCs (Fig. 7A). Cholesterol treatment restored the presence of N-cadherin, DDR1, and associated catenins in lipid raft fractions of DDR1+/+ VSMCs (Fig. 7A). In DDR1−/− VSMCs MβCD treatment did not affect N-cadherin or catenin levels in lipid rafts (Fig. 7B). However, cholesterol treatment actually increased the amount of N-cadherin and its associated catenins in the lipid raft fractions of DDR1−/− VSMCs (Fig. 7B), but this was not accompanied by restoration of N-cadherin at morphologic cell–cell contacts (Fig. 6B).

### N-cadherin junction staining was reduced after DDR1 knockdown, and rescued after DDR1b overexpression

**Fig. 8.**
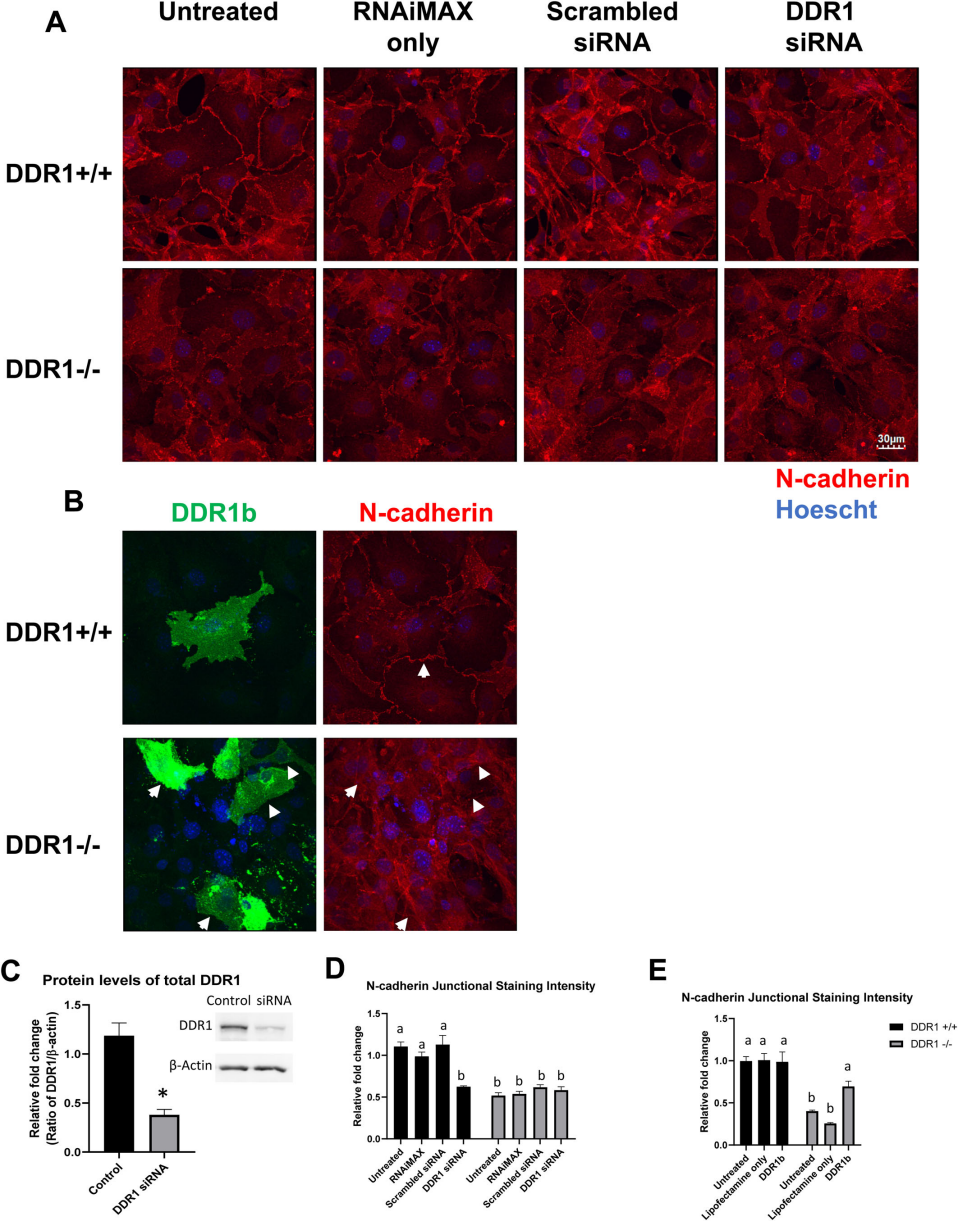
**N-cadherin junction staining was reduced after DDR1 knockdown, and rescued after DDR1b overexpression.** (A) Immunofluorescence of DDR1+/+ and DDR1−/− untreated VSMCs, cells transfected with RNAiMAX only, or scrambled siRNA or DDR1 siRNA, then fixed and stained for N-cadherin (red; 610921 BD Biosciences). Nuclei are counterstained with Hoechst (blue). (B) DDR1+/+ and DDR1−/− VSMCs were transfected with DDR1b plasmid DNA, then fixed and stained for DDR1 (green; D1G6 Cell Signaling Technology) and N-cadherin (red; 610921 BD Biosciences). Nuclei are counterstained with Hoechst (blue). Green staining identifies transfection and expression of the DR1b plasmid. Arrows point to junctional N-cadherin staining in VSMCs expressing exogenous DDR1b. (C) DDR1 protein levels in cells transfected with control or DDR1 siRNA. Experiment was repeated three times. Data are plotted as mean±s.e.m. Student’s *t*-test was performed. (D) Relative fold change in the intensity of junctional N-cadherin staining in DDR1+/+ and DDR1−/− VSMCs after siRNA treatment. Designation with the same letter represents values not significantly different. Experiment was repeated three times. Data are plotted as mean±s.e.m. One-way ANOVA followed by Holm-Sidak test was performed. (E) Relative fold change in the intensity of junctional N-cadherin staining in DDR1+/+ and DDR1−/− VSMCs after DDR1b transfection. Designation with the same letter represents values not significantly different. Experiment was repeated three times. Data are plotted as mean±s.e.m. One-way ANOVA followed by Holm-Sidak test was performed.

To ensure the disruption in N-cadherin cell–cell contacts observed in DDR1−/− VSMCs was specifically due to DDR1 deletion instead of possible confounding effects in the global DDR1 knockout model, DDR1+/+ and DDR1−/− VSMCs were treated with DDR1 siRNA and stained for N-cadherin. DDR1 levels were decreased ∼70% in cells treated with DDR1siRNA (Fig. 8C). In untreated cells and cells treated with RNAiMAX (vehicle) or scrambled siRNA, N-cadherin contacts were arranged in a zipper conformation around the periphery of DDR1+/+ VSMCs, while N-cadherin staining was more diffuse in the cytoplasm of DDR1−/− VSMCs (Fig. 8A). DDR1 silencing reduced the junctional staining of N-cadherin and its zipper-like conformation in DDR1+/+ VSMCs, but had very little effect on cytoplasmic N-cadherin staining in DDR1−/− VSMCs (Fig. 8A). Quantification revealed that junctional staining for N-cadherin was reduced by 43% after DDR1 siRNA treatment in DDR1+/+ VSMCs, but the low level of junctional staining was not significantly affected by DDR1 siRNA treatment in DDR1−/− VSMCs (Fig. 8D).

To further confirm the direct effect of DDR1 on N-cadherin cell–cell contact formation in VSMCs, DDR1+/+ and DDR1−/− VSMCs were transfected to overexpress full length DDR1b plasmid. Cells were immunostained with an antibody against DDR1 to detect successful transfection of the DDR1b plasmid (green, intense stain due to overexpression of high levels of DDR1) followed by immunostaining for N-cadherin (red). Fig. 8B, lower panels shows several DDR1−/− cells overexpressing DDR1b (green), and the establishment of N-cadherin stained cell–cell junctions (red) (Fig. 8B, arrows). Quantification of the immunofluorescent staining revealed that expression of DDR1b increased junctional N-cadherin by 73% in DDR1−/− VSMCs (Fig. 8E). By contrast, DDR1b transfection did not further increase N-cadherin junctional staining in DDR1+/+ VSMCs (Fig. 8E).

### The cytoskeleton was disrupted in DDR1−/− VSMCs

**Fig. 9.**
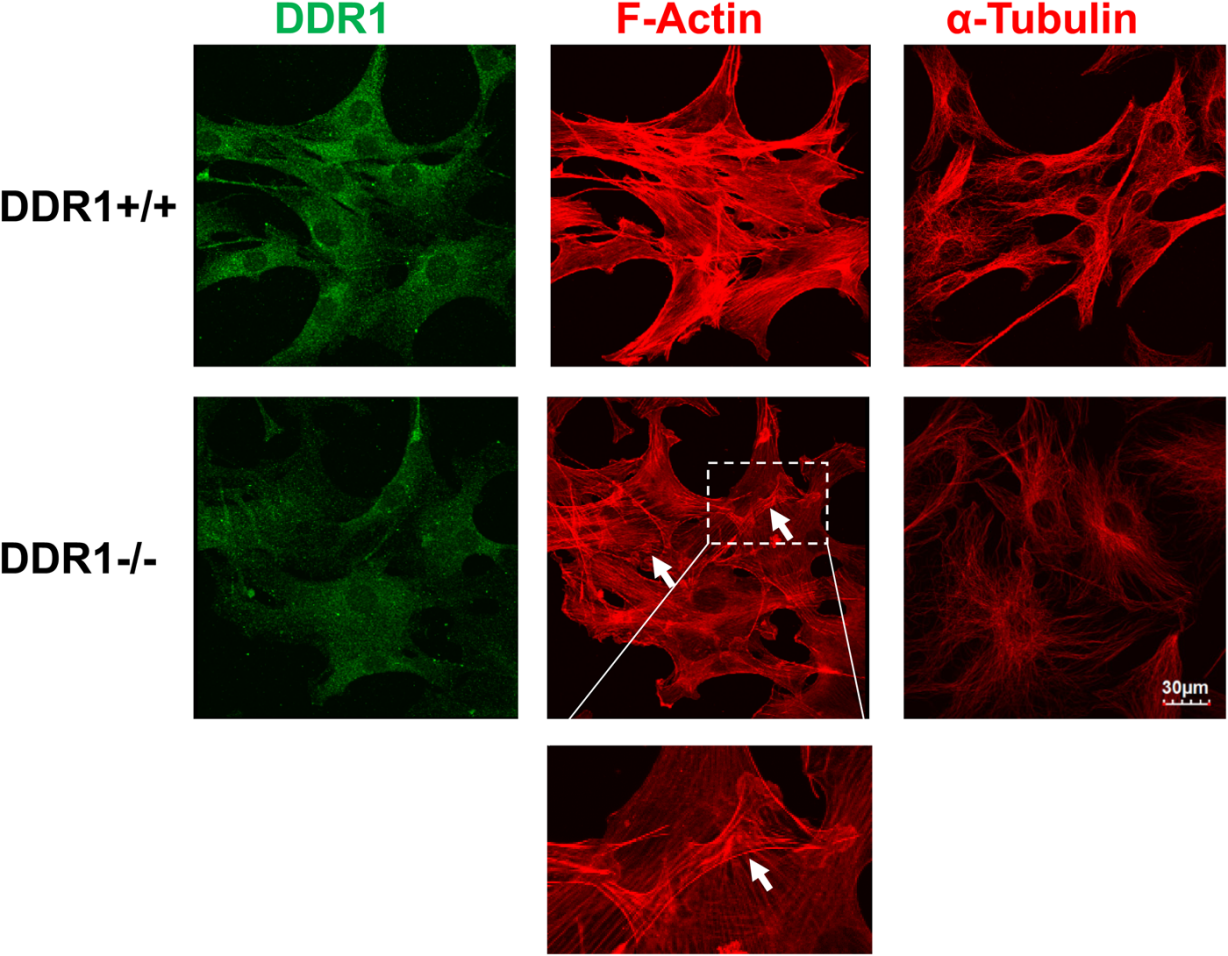
**The cytoskeleton was disrupted in DDR1−/− VSMCs.** Immunofluorescence of DDR1+/+ and DDR1−/− VSMCs fixed and stained for DDR1 (green; D1G6 Cell Signaling Technology), F-actin (red; Alexa 568 phalloidin), α-tubulin (red; ab52866 Abcam). Arrows identify areas of membrane ruffling and overlap in DDR1−/− VSMCs. Dotted lines enclose an area showing a membrane ruffle which is shown at higher magnification below.

Cell contacts are stabilized by anchoring to the cytoskeleton, but our previous studies have revealed actin and microtubule disruption in DDR1−/− VSMCs. We stained DDR1+/+ and DDR1−/− VSMCs for F-actin and α-tubulin to visualize the cytoskeleton. Immunostaining cells with DDR1 antibody revealed some non-specific staining in the DDR1−/− cells due to antibody cross-reacting with other proteins (Fig. 9), as has been noted previously on western blots. DDR1−/− VSMCs showed weak F-actin staining, with a dramatic decrease in the number of stress fibres, a reduction in cortical actin, and increased membrane ruffles that overlapped at contact sites between neighbouring cells (Fig. 9, arrows and inset panel). Microtubules were less abundant and less aligned in DDR1−/− VSMCs (Fig. 9). Cells were treated with cytochalasin D to inhibit actin polymerization and determine whether this affected N-cadherin cell–cell contacts. DDR1+/+ VSMCs showed disruption of N-cadherin cell–cell contacts after cytochalasin D treatment compared to untreated cells (data not shown). By contrast, while cytochalasin D had a disruptive effect on the actin cytoskeleton of DDR1−/− VSMCs, it did not lead to disruption of the cytoplasmic N-cadherin staining in DDR1−/− VSMCs compared to untreated cells (data not shown).

## DISCUSSION

This study was conducted to further our understanding of the molecules and structures in VSMCs that are important for proper N-cadherin junction establishment, and to determine whether DDR1 associates with and promotes the expression or function of N-cadherin. Our results show that DDR1 regulates N-cadherin junction stability and localization to lipid rafts in VSMCs. Knockout or knockdown of DDR1 in VSMCs resulted in disruption of N-cadherin contacts. Analysis of lipid raft fractions revealed decreases in the amounts of N-cadherin and associated catenins in DDR1−/− compared to DDR1+/+ VSMCs. Importantly, transfection of DDR1−/− cells with full-length DDR1b rescued the KO phenotype and promoted the formation of N-cadherin junctions.

N-cadherin is the most abundant cell–cell adhesion molecule expressed by VSMCs, and it plays an important role in regulating cellular functions including migration, proliferation, and survival (Lyon et al., 2010; Sabatini et al., 2008; Uglow et al., 2003). Research from our lab has shown that wound edge VSMCs after mechanical wounding *in vitro* displayed a polarized posterior-lateral distribution of N-cadherin cell–cell contacts, which was required for front polarization of the microtubule organizing centre, anterior positioning of hyper-stabilized microtubules to facilitate membrane transport, activation of Cdc42 at the leading edge, inhibition of GSK3β at the posterior-lateral edge, and directional migration into the wound (Sabatini et al., 2008). The effects of N-cadherin on Rho GTPases were also found in C2C12 myoblasts where the establishment of N-cadherin contacts inhibited Cdc42 and Rac1 activity as well as filopodia and lamellipodia formation (Charrasse et al., 2002). In VSMCs, downregulation and disruption of N-cadherin cell–cell contacts were associated with increased proliferation caused by the translocation of β-catenin into the nucleus to activate transcription (Uglow et al., 2003). Furthermore, inhibiting N-cadherin function and abolishing N-cadherin expression increased apoptosis in VSMCs and greatly impacted cell survival (Lyon et al., 2010). Overall, these findings suggest that the ability to establish proper N-cadherin cell–cell contacts is crucial to VSMC function.

While interactions between DDR1 and N-cadherin have not been previously investigated in VSMCs, both molecules were found in separate studies to be upregulated in the neointima after mechanical injury of the carotid arteries coincident with the time course of active proliferation and migration of these cells (Hou et al., 2001; Jones et al., 2002). Upon deletion of DDR1 in mice, VSMC migration after denuding injury was reduced, mice developed smaller atherosclerotic plaques and DDR1−/− VSMCs exhibited reduced migration *in vitro* (Franco et al., 2008; Hou et al., 2001). VSMC migration and neointimal formation were also impaired after the functional inhibition of N-cadherin (Lyon et al., 2010; Sabatini et al., 2008). Our current results show for the first time that DDR1 and N-cadherin interact in VSMCs, and suggest that DDR1 influences the localization and stability of cell adhesion junctions, identifying a pathway whereby matrix and cell adhesions coordinate to regulate cell migration and proliferation.

Since DDR1 has been shown to associate with E-cadherin in epithelial cells to enhance the stabilization of E-cadherin-based junctions (Eswaramoorthy et al., 2010; Yeh et al., 2011), we investigated whether DDR1 could associate with N-cadherin in VSMCs. DDR1 and N-cadherin co-immunoprecipitated with each other, suggesting that the two molecules exist in physical proximity to each other in VSMCs, likely in the lipid rafts. In epithelial cells DDR1 has been shown to upregulate N-cadherin mRNA expression and protein synthesis during the process of EMT (Miao et al., 2013). However, in VSMCs, total protein levels of N-cadherin were similar between DDR1+/+ and DDR1−/− VSMCs, arguing against a role for DDR1 in regulating N-cadherin expression and synthesis. This suggests that DDR1-mediated regulation of N-cadherin junctions in VSMCs occurs through a different mechanism. Since there was disruption of N-cadherin contacts on the cell membrane in DDR1−/− VSMCs, we measured total membrane levels of N-cadherin protein in DDR1+/+ and DDR1−/− VSMCs. However, we did not detect a difference between the two genotypes. Another factor that contributes to N-cadherin contact formation and stabilization is its association with catenin molecules. However, when we immunoprecipitated N-cadherin from DDR1+/+ and DDR1−/− VSMCs, we detected similar levels of α-, β-, γ-, and p120-catenin associated with N-cadherin, suggesting that the disruption of N-cadherin contacts in DDR1−/− VSMCs is unlikely due to altered binding of catenin molecules during junctional complex formation.

Lipid rafts comprise specialized microdomains within the cell membrane and serve as an important nexus for cell signalling; cell–cell and cell–matrix adhesion molecules are known to concentrate in rafts. Previous reports in C2C12 myoblasts showed that concentration of N-cadherin in lipid rafts decreased lateral diffusion and mobility within the plasma membrane, and this stabilizing effect occurred without changes in the total level of N-cadherin in the membrane (Causeret et al., 2005). To determine how DDR1 might influence N-cadherin-mediated adhesion in VSMCs, we studied the localization of N-cadherin junctional complexes to lipid rafts in DDR1+/+ and DDR1−/− cells. Performing Triton extraction, co-immunostaining of N-cadherin and GM1, fractionation over a sucrose density gradient, and cholesterol depletion and rescue, we showed that the N-cadherin and catenin junctional complex proteins were associated with lipid rafts, and that this association was reduced in DDR1−/− VSMCs. Moreover, we present convincing evidence that DDR1 also localizes to lipid rafts. Therefore, we propose that the close association between DDR1 and N-cadherin in lipid rafts of VSMCs helps cluster N-cadherin junctional complex into lipid rafts, immobilizing and stabilizing contacts, and in turn stabilizing DDR1.

Another important factor in contact stabilization is the association with the actin cytoskeleton, which can help anchor and cluster junctional complexes. In DDR1−/− VSMCs actin staining intensity and stress fibres were reduced, and microtubule organization was altered. Both cytoskeletal structures play important roles in adherens junction formation. Transport along microtubules is important for trafficking of N-cadherin to the plasma membrane where it can form cell–cell contacts (Mary et al., 2002). N-cadherin staining in the cell cytoplasm was increased in DDR1−/− cells, so it is possible that N-cadherin trafficking was altered. It is also possible that recycling of N-cadherin in endocytotic vesicles was impaired. Another explanation is that in the absence of DDR1, N-cadherin in the plasma membrane cannot localize to lipid rafts and/or anchor to the actin cytoskeleton, therefore adherens junctions do not mature and stabilize. This was further supported by our data demonstrating the disruptive effect of cytochalasin D on N-cadherin contacts in DDR1+/+ VSMCs.

In conclusion, we have shown that DDR1 facilitates the stabilization of N-cadherin cell–cell contacts in VSMCs. Specifically, we have shown that DDR1 associates with N-cadherin, and the deletion of DDR1 disrupted N-cadherin cell–cell contacts in VSMCs through a reduction in lipid raft association as well as effects on the cytoskeleton. The molecular mechanisms underlying N-cadherin-mediated adhesion in VSMCs are important in regulating the migratory and proliferative behaviour of VSMCs, which are essential processes contributing to atherosclerotic plaque formation and restenosis after angioplasty, stenting or in vein grafts.

## MATERIALS AND METHODS

All reagents were obtained from Sigma Chemical unless stated otherwise.

### Cell culture

Animal experiments were performed in accordance with the guidelines of the Canada Council on Animal Care, with the approval of the University of Toronto Faculty of Medicine Animal Care Committee. Primary mouse VSMCs from the carotid arteries of male and female DDR1+/+;C57Bl6 and DDR1−/−;C57Bl6 mice were used between passages 5 and 9. VSMCs were grown in Dulbecco's Modified Eagles Medium (DMEM) supplemented with 10% foetal bovine serum, and 2% penicillin, streptomycin and amphotericin (GIBCO BRL, Life Technologies Inc., Rockville, MD, USA) in humidified 95% air and 5% carbon dioxide in an incubator at 37°C.

### Membrane fractionation

DDR1+/+ and DDR1−/− VSMCs were plated on 150-mm plastic tissue culture plates at 25,000 cells/cm^2^ and grown to confluence for 4–5 days then serum starved overnight. Cells were then rinsed with ice-cold PBS, lysed by scraping on ice into 600 μl sucrose buffer (250 mM sucrose, 20 mM HEPES, 10 mM KCl, 2 mM MgCl_2_, 1 mM EDTA, 1 mM EGTA), supplemented with 1 mM DTT, 100 µM vanadate, 100 µM phenylmethylsulfonyl fluoride (PMSF), protease inhibitor cocktail (Catalogue No. 11836170001, Roche Applied Science, Germany), and homogenized by passing the cell lysate through a 30G needle ten times using a 1 ml syringe. A portion was saved as the whole cell lysate. Cells were left on ice for 20 min to lyse and then centrifuged at 800 ***g*** for 5 min at 4°C to pellet nuclei. The supernatant was centrifuged again at 10,000 ***g*** for 5 min to pellet mitochondria. Finally, the supernatant was ultracentrifuged at 100,000 ***g*** for 45 min to pellet membrane. The resulting supernatant was saved as the cytoplasmic portion, and the pellet was resuspended in TBS+0.1% SDS and saved as the membrane portion. All lysate portions were dissolved in Laemmli buffer with 5% β-mercaptoethanol, boiled for 5 min, and analysed by western blotting.

### Co-immunoprecipitation

DDR1+/+ and DDR1−/− VSMCs were plated on 150-mm plastic tissue culture plates at 25,000 cells/cm^2^ for 4–5 days to near confluence then serum starved overnight. Cells were lysed with 1× cell lysis buffer (#9803, Cell Signaling Technology, Inc., MA, USA, 20 mM Tris-HCl, pH 7.5, 150 mM NaCl, 1 mM Na_2_EDTA, 1 mM EGTA, 1% Triton, 2.5 mM sodium pyrophosphate, 1 mM beta-glycerophosphate, 1 mM Na_3_VO_4_, 1 µg/ml leupeptin) supplemented with 1 mM PMSF, 20 mM NaF, and protease inhibitor cocktail, sonicated or passed through a 30 G needle five times, rotated at 4°C for 15 min to lyse, and centrifuged at 14,000 ***g*** for 15 min at 4°C. Supernatant was incubated with anti-DDR1 antibody (1:100, D1G6, #5583, Cell Signaling Technology Inc.) or anti-N-cadherin antibody (1:100, ab18203, Abcam) overnight at 4°C with constant rotation. Afterwards, Protein A agarose beads (#9863, Cell Signaling Technology Inc.) were added to the lysate to incubate for 3 h at 4°C with constant rotation. Afterwards beads were washed three times with lysis buffer. Beads and total cell lysates were denatured in Laemmli buffer with 5% β-mercaptoethanol, boiled for 5 min, and subjected to western blotting.

### Triton extraction

DDR1+/+ and DDR1−/− VSMCs were plated on 18 mm round glass coverslips at 20,000–30,000 cells/cm^2^ and grown to near confluence, serum starved overnight, treated with 1% Triton in ice-cold 10 mM PIPES, pH 6.8, 50 mM NaCl, 3 mM MgCl_2_, 300 mM sucrose, and 1 mM PMSF for 5 min, then fixed in 4% paraformaldehyde and stained for N-cadherin.

DDR1+/+ and DDR1−/− VSMCs were grown on 100-mm plastic tissue culture plates to near confluence and lysed for 15 min at 4°C with constant rotation in 1× cell lysis buffer (#9803, Cell Signaling Technology Inc.), supplemented with 1 mM PMSF, 20 mM NaF, and protease inhibitor cocktail (Roche). Cell lysate was sonicated and centrifuged at 13,200 ***g*** for 15 min at 4˚C. Supernatant and pellet was saved as triton-soluble and triton-insoluble portion, respectively. Lysates were dissolved in Laemmli buffer with 5% β-mercaptoethanol, boiled for 5 min, and subjected to western blotting.

### GM1 staining

DDR1+/+ VSMCs were plated on 22 mm×22 mm glass coverslips at 20,000–30,000 cells/cm^2^ and grown to near confluence and incubated with 15 μg/ml of Alexa488-conjugated cholera toxin subunit B (C34775, Life Technologies Inc.), which binds to GM1 enriched in lipid rafts, for 15 min on ice. Afterwards, cells were fixed in 4% paraformaldehyde and stained for N-cadherin.

### Lipid raft isolation

DDR1+/+ and DDR1−/− VSMCs were plated on 150-mm plastic tissue culture plates at 25,000 cells/cm^2^ and grown to near confluence and serum starved overnight, rinsed with ice-cold PBS, and lysed in 25 mM Tris, pH 7.5, 150 mM NaCl, and 5 mM EDTA containing 1% Triton, 200 μM NaF, 100 μM PMSF, 100 μM sodium orthovanadate, and protease inhibitor tablet. Cell lysate was rotated at 4°C for 30–60 min, then passed ten times through a 30 G needle. Equal concentrations and volumes of cell lysate were mixed with 80% sucrose to obtain 40% final sucrose concentration, overlaid with 30% sucrose followed by 5% sucrose for a final volume of 4.5 ml, and centrifuged at 200,000 ***g*** at 4°C for 16–18 h. From the top, 500 μl fractions were collected, dissolved in 2× Laemmli buffer with 5% β-mercaptoethanol, boiled for 5 min, and subjected to western blotting.

### Cholesterol oxidase treatment

DDR1+/+ and DDR1−/− VSMCs were plated on 18 mm round glass coverslips at 20,000–30,000 cells/cm^2^ and grown on to near confluence and serum starved overnight, treated with 1 or 2 units of cholesterol oxidase (C8649, Sigma-Aldrich) for 1 or 2 h at 37°C. Afterwards, cells were fixed in 4% paraformaldehyde and stained for N-cadherin.

### Methyl-β-cyclodextrin treatment and cholesterol rescue

DDR1+/+ and DDR1−/− VSMCs were plated at 20,000–30,000 cells/cm^2^ on 18 mm round glass coverslips and grown to near confluence and serum starved overnight, treated with 1 mM of methyl-β-cyclodextrin (MβCD, C4555, Sigma-Aldrich) for 30 min at 37°C. Afterwards, cells were treated with or without 1 mM of MβCD-cholesterol complex (C4951, Sigma-Aldrich) for 30 min or 1 h at 37°C. Cells were then fixed in 4% paraformaldehyde and stained for N-cadherin.

### DDR1 siRNA transfection

DDR1+/+ and DDR1−/− VSMCs were plated at 30,000 cells/cm^2^ and grown to 90% confluence and transfected with or without RNAiMAX only (Thermo Fisher Scientific), scrambled siRNA (AM4635, Thermo Fisher Scientific), or DDR1-specific siRNA (AM16704, ID 159939, Ambion, Thermo Fisher Scientific) using Lipofectamine RNAiMAX Transfection Reagent (Cat No. 13778075, Thermo Fisher Scientific) according to the manufacturer's instructions. Briefly, lipofectamine RNAiMAX was mixed with siRNA, allowed to incubate at room temperature for 5 min, and added to the cells for a final siRNA concentration of 10 nM. Cells were cultured for 24 h, fixed in 4% paraformaldehyde, and stained for N-cadherin. Parallel cultures were lysed and prepared for western blots to measure the efficiency of DDR1 knockdown.

### DDR1b transfection

Plasmid containing full-length DDR1b isoform (a gift from the late Dr Wolfgang Vogel) was transformed into competent DH5-α *E. coli* according to the manufacturer's instructions (C2987H; New England Biolabs). Plasmid purification was performed using Maxi Prep DNA isolation kit according to the manufacturer's instructions (K210026; Invitrogen). Plasmids containing DDR1b or empty vector were transfected into DDR1+/+ and DDR1−/− VSMCs using Lipofectamine-3000 according to the manufacturer's instructions (L3000015; Thermo Fisher Scientific). Briefly, cells were plated at 30,000 cells/cm^2^ in six-well plates and grown to 90% confluence. Lipofectamine 3000 (3.75 μl/well) and plasmid DNA (2.5 μg/well) were mixed, allowed to incubate at room temperature for 5 min, and added directly to cells. Cells were cultured for 24 h, fixed in 4% paraformaldehyde, and stained for DDR1 and N-cadherin.

### Cytochalasin D treatment

DDR1+/+ and DDR1−/− VSMCs were grown on 18 mm round glass coverslips to near confluence and serum starved overnight, treated with 1 μg/ml of cytochalasin D (C2618, Sigma-Aldrich) for 1 h at 37°C. Afterwards, cells were fixed in 4% paraformaldehyde and stained for N-cadherin.

### Immunofluorescent staining and confocal microscopy

Cells were fixed with 4% paraformaldehyde for 5 min or 100% ice-cold methanol for 10 min, rinsed in PBS, incubated with 0.2% Triton X-100 in PBS for 5 min, and washed in PBS three times at 5 min intervals. The coverslips were incubated with mouse monoclonal anti-N-cadherin (1:250, 610921, BD Biosciences), rabbit anti-DDR1 (1:700, D1G6, Cell Signaling Technology Inc.), or rabbit anti-α-tubulin (1:250, ab52866, Abcam), primary antibody for 1 h at room temperature. The coverslips were washed three times with PBS at 5-min intervals. Secondary antibodies donkey anti-mouse Alexa 488, goat-anti-rabbit Alexa 488, donkey-anti-mouse Alexa 568, goat-anti-rabbit Alexa 568 (1:200, Invitrogen), Alexa 568-conjugated phalloidin (1:100, Invitrogen), and Hoechst 33342 (1:5000, Lonza) were applied to washed coverslips and incubated for 20 min. The coverslips were then washed again three times with PBS at 5 min intervals followed by dipping in distilled water. Prolong Gold (Invitrogen) was used for mounting of coverslips to glass slides for microscopic observation. Coverslips were examined using the 60× objective of a laser scanning confocal microscope (FluoView 1000 Laser Scanning Confocal Microscope, Olympus IX81 Inverted Microscope, Olympus Co. Canada). Serial optical sections were taken at 0.5-μm thickness to include the entire thickness of the cells. Z-stacks were analysed and displayed for each experiment.

### Western blot

Proteins were separated on SDS-PAGE gels at 70–90 V for 1.5–2 h at room temperature in running buffer (2.5 mM Tris, 25 mM glycine, 0.1% SDS), and transferred overnight at 35 V at 4°C in transfer buffer (2.5 mM Tris 19.2 mM glycine) to polyvinylidene fluoride membranes. Immunoblotting was performed with rabbit anti-DDR1 antibody (D1G6, 1:1000, Cell Signaling Technology), rabbit anti-N-cadherin antibody (ab76057, 1:5000, Abcam), rabbit anti-β-catenin antibody (8480, 1:1000, Cell Signaling Technology), rabbit anti-γ-catenin antibody (2309, 1:1000, Cell Signaling Technology), rabbit anti-p120-catenin antibody (ab92514, 1:1000, Abcam), rabbit anti-flotillin-2 antibody (3436, 1:1000, Cell Signaling Technology), rabbit anti-caveolin-1 antibody (D46G3, #3267, 1:1000, Cell Signaling Technology), rabbit anti-β-actin antibody (ab8224, 1:1000, Abcam), followed by HRP-conjugated anti-mouse or anti-rabbit secondary antibody (1:10,000, Jackson ImmunoResearch Laboratories, Inc. USA), or HRP-conjugated anti-GAPDH antibody (ab9482, 1:5000, Abcam). Blots were stripped and reprobed for different proteins. Immunoreactive bands were visualized using Westar SuperNova Chemiluminescence Detection Kit (Cyanagen, Bologna, Italy) or Western Lightning Plus-ECL (PerkinElmer Inc.) and the image was captured using MicroChemi bio-imaging system (Froggabio, Toronto, Canada) or ChemiDoc Touch Imaging System (Bio-Rad Laboratories Inc.). Western blots were quantified by measuring the intensity of bands (ImageJ, NIH), and normalizing to β-actin (Figs 2A, 4D,E, 8C), flotillin-2 (Fig. 2D,E), N-cadherin (Fig. 3C), or caveolin-1 (Fig. 5D). To enable statistical analysis, for each experiment one control sample was arbitrarily chosen and set to a value of one. The value of every other control and experimental sample was then calculated relative to that sample, and the mean of the control and experimental groups and standard error on the means were determine and plotted. This enabled us to assess the variance in the control and experimental sample groups, and perform statistical analysis on the data.

### Image processing and analysis

Quantitative analysis of the junctional intensity of N-cadherin was performed in ImageJ software (NIH) with the line scan functions by drawing straight lines orthogonal to, and centred on, randomly selected homotypic junctions. Optical Z-stacks (0.5-μm intervals) were acquired to correct for cell heights and to focus on all junctions analysed. The maximum and minimum pixel intensities along the selected lines were recorded, and the minimum pixel intensities were subtracted from the maximum pixel intensities as background fluorescence. A total of 20–30 junctions were analysed for each individual experiment, and at least three independent experiments were performed.

### Statistical analysis

Statistical analysis was performed using GraphPad Prism. A value of *P*<0.05 was considered significant. Student's *t*-test was used for pair-wise comparisons. One-way ANOVA followed by Holm-Sidak test was used for comparison between multiple groups.
